# EGCG Enhances Cisplatin Sensitivity by Regulating Expression of the Copper and Cisplatin Influx Transporter CTR1 in Ovary Cancer

**DOI:** 10.1371/journal.pone.0125402

**Published:** 2015-04-30

**Authors:** Xuemin Wang, Pan Jiang, Pengqi Wang, Chung S. Yang, Xuerong Wang, Qing Feng

**Affiliations:** 1 Department of Nutrition and Food Hygiene, Key Laboratory of Toxicology, School of Public Health, Nanjing Medical University, Nanjing, Jiangsu, China; 2 Department of Chemical Biology, Center for Cancer Prevention Research, Ernest Mario School of Pharmacy, Rutgers, the State University of New Jersey, Piscataway, New Jersey, United States of America; 3 Department of Pharmacology, School of Basic Medical Science, Nanjing Medical University, Nanjing, Jiangsu, China; 4 Beijing Research Institute for Nutritional Resources, Beijing, China; Garvan Institute of Medical Research, AUSTRALIA

## Abstract

Cisplatin is one of the first-line platinum-based chemotherapeutic agents for treatment of many types of cancer, including ovary cancer. CTR1 (copper transporter 1), a transmembrane solute carrier transporter, has previously been shown to increase the cellular uptake and sensitivity of cisplatin. It is hypothesized that increased CTR1 expression would enhance the sensitivity of cancer cells to cisplatin (cDDP). The present study demonstrates for the first time that (-)-epigallocatechin-3-gallate (EGCG), a major polyphenol from green tea, can enhance CTR1 mRNA and protein expression in ovarian cancer cells and xenograft mice. EGCG inhibits the rapid degradation of CTR1 induced by cDDP. The combination of EGCG and cDDP increases the accumulation of cDDP and DNA-Pt adducts, and subsequently enhances the sensitivity of ovarian cancer SKOV3 and OVCAR3 cells to the chemotherapeutic agent. In the OVCAR3 ovarian cancer xenograft nude mice model, the combination of the lower concentration of cDDP and EGCG strongly repressed the tumor growth and exhibited protective effect on the nephrotoxicity induced by cisplatin. Overall, these findings uncover a novel chemotherapy mechanism of EGCG as an adjuvant for the treatment of ovarian cancer.

## Introduction

Ovarian cancer is the seventh most common cancer (estimated age-standardized incidence and mortality) in women worldwide and is one of the leading causes of mortality among gynecological malignancies [1]. In recent decades, even though new therapeutic strategies have been developed, surgery and platinum-based chemotherapy are still the standard treatments for ovarian cancer [2].

Cisplatin (cis-diamminedichloroplatinum, cDDP) is one of the first-line chemotherapeutic agents in the treatment of ovarian cancer. cDDP exerts its cytotoxic effect predominantly by formatting an intra-strand cross-linking on DNA that blocks transcription and DNA replication, resulting in cell apoptosis [3]. However, drug resistance is an important limitation in the clinical application of cDDP. Mechanisms of cDDP resistance are complicated, including decreased drug uptake, increased drug efflux, increased DNA damage repair and alterations in apoptotic signaling pathways [4]. Recent studies suggested that copper transporters not only are involved in copper homeostasis but also regulate the cellular pharmacology and sensitivity to platinum-based agents [5, 6].

The family of copper transporters consists of copper transporters and copper transporting phosphorylated ATPase (ATP7A and ATP7B). The former includes transmembrane solute carrier transporter CTR1 (encoded by *SLC31A1*) and CTR2 which regulate the influx of platinum-containing agents. CTR1 contains 190 amino acids and has three transmembrane domains. The N-terminal extracellular domain is rich in methionine and histidine, and is essential for transporting copper [7, 8]. Several studies indicated that CTR1 is a major regulator of the efficacy of platinum drugs *in vitro* and *in vivo* [9, 10]. Over-expression of CTR1 was found to sensitize cells to platinum drugs by increasing drug uptake [11, 12]; whereas knock-down of CTR1 rendered cells resistant to these agents in yeast and several mammalian cells [13–15]. CTR1 could be rapidly degraded by cDDP, even at a low concentration (2μM) [15]. This process involves ubiquitination and proteasomal degradation and requires the copper chaperone antioxidant protein 1 (ATOX1) [16]. Unlike CTR1, CTR2 decreases cellular cDDP accumulation and sensitivity to cDDP of several types of tumors [17, 18]. Knock-down of CTR2 leads to increased concentration of cDDP and this effect is opposite to knock-down of CTR1 [19]. ATP7A and ATP7B facilitate the sequestration and export of platinum-containing agents and are two promising chemoresistance markers for cDDP in various solid tumors [20, 21].

(-)-Epigallocatechin-3-gallate (EGCG) is the most abundant and active polyphenol found in green tea and has been extensively studied in cancer prevention and therapy [22,23]. It has been found that EGCG has anti-cancer effect in several cancer types including ovarian cancer.The anti-cancer effect of EGCG may involve its inhibition of cancer process during the initiation, progression and metastasis [24].

There is a large body of evidence suggests that EGCG enhances the effect of conventional cancer therapies in multiple cell models [25]. In the case of the combination of EGCG and cDDP, EGCG can enhance the sensitivity of cDDP via multiple mechanisms, including up-regulating caspase-9a [26], potentiating G2/M arrest and up-regulating p21 [27], as well as inducting of apoptosis through enhanced intracellular H_2_O_2_ generation [28]. EGCG treatment also inhibits telomerase expression and makes cells more sensitive to cDDP [29]. A more recent study found that EGCG suppressed ABCC2 and ABCG2 transporter genes, then augmented the efficacy of cDDP [30]. As a chemosensitizer, EGCG regulates microvasculature and microenvironment and improved cDDP sensitivity by rebalancing Ang-1 and Ang-2 [31]. In lung cancer cells, EGCG could enhance the efficacy of cDDP by down-regulating hsa-miR-98-5p [32].

Although the mechanisms of action of EGCG in combination with cDDP have been intensively studied, there is no research focused on the uptake of cDDP. Here, we investigated whether EGCG could modulate the copper transporters involved in the uptake and efflux of cDDP *in vitro* and *in vivo*. We analyzed the effect of EGCG in combination with cDDP on cells sensitivity to cDDP, the accumulation of cDDP and DNA-cDDP adducts, and the expression of copper transporters in ovarian cancer cells. Interestingly, we made a novel observation that EGCG induced the expression of CTR1 *in vivo and in vitro*. The findings provides experimental evidence for considering the application of EGCG as an adjuvant in ovarian cancer therapy.

## Materials and Methods

### Cell culture and reagents

Human ovarian cancer OVCAR3, SKOV3 cells and human embryonic kidney HEK-293T cells were obtained from Chinese Academy of Sciences Committee on Type Culture Collection Cell Bank (Shanghai, China). All these cells were cultured in Dulbecco's Modified Eagle Medium (DMEM, GIBCO, Carlsbad, CA, USA) supplemented with 10% heat-inactivated fetal bovine serum (GIBCO, Carlsbad, CA, USA), and 100U/ml penicillin and 100mg/ml streptomycin. Cells were incubated at 37°C in a humidified atmosphere of 5% CO_2_. EGCG and cDDP were purchased from Sigma-Aldrich (St. Louis, MO, USA).

### MTT assay

MTT assay was used to evaluate cell viability. Cells were seeded in a 96-well plate at a density of 2 × 10^3^ per well and allowed to attach overnight. Then EGCG alone or in combination with cDDP was administrated in a final volume of 200 μl of medium. After treatment for 24 h or 48 h, the cells were incubated at 37°C with 20 μl of MTT solution (5 mg/ml,Amresco, OH, USA) for 4 h. The MTT formazan crystal was then dissolved in 150 μl DMSO (Lingfeng, Shanghai, China), and a microplate reader (Tecan, Mannedorf, Switzerland) was used to measure the absorbance at 490 nm.

### Colony formation assay

After indicated treatment, the cells (2 × 10^2^) were seeded in a 6-well plate and changed to a fresh medium every 3 days. After three weeks, visible colonies were fixed and stained with crystal violet staining solution (Beyotime, Shanghai, China).

### Hoechst 33258 staining

The cells were seeded in a 6-well plate. After incubation for 24 h, EGCG or cDDP was added alone or in combination for 48 h. Then the cells were fixed in 4% paraformaldehyde for 10 min, and washed twice with PBS. After being stained with 0.5 ml of Hoechst 33258 (Beyotime, Shanghai, China) for 5 min, the cells were again washed twice with PBS. The stained nuclei were observed under an inverted fluorescence microscopy (Olympus, Japan).

### Measurement of platinum (Pt) accumulation in cells

Whole-cell Pt content was measured by a Perkin-Elmer Element 2 inductively coupled plasma mass spectrometry (ICP-MS) according to previously reported methods [17]. For measurement of Pt in DNA, the DNA was extracted using DNAzol (Invitrogen, Carlsbad, CA, USA) according to the manufacturer’s protocol. For normalization, the concentration of DNA was measured using a Nanodrop 2000 spectrophotometer (Thermo Scientific, Wilmington, DE, USA). The DNA samples were then digested in 5% nitric acid and measured by ICP-MS.

### siRNA transfection

Human CTR1 siRNA or siRNA control (RiboBio, Guangzhou, China) were transfected into OVCAR3 and HEK-293T cells in a 96-well plate using Lipofectamine 2000 reagent (Invitrogen, Carlsbad, CA, USA), following the manufacturer’s instructions.Si-h-CTR1 sequences are listed below:

si-h-CTR1 001: 5’ GAUCAAUACAGCUGGAGAA dTdT 3’ (forward)

3’dTdT CUAGUUAUGUCGACCUCUU 5’ (reverse);

si-h-CTR1 002: 5’GGAAGAAGGCAGUGGUAGU dTdT 3’ (forward)

3’ dTdT CCUUCUUCCGUCACCAUCA 5’ (reverse);

si-h-CTR1 003: 5’ CUACUUUGGCUUUAAGAAU dTdT 3’ (forward)

3’dTdT GAUGAAACCGAAAUUCUUA 5’ (reverse).

### Reverse Transcription Polymerase Chain Reaction (RT-PCR)

Total RNA was isolated from cells using the RNAiso Plus (TaKaRaBioTechnology, Dalian, China) following the manufacturer’s protocol. The concentration of total RNA was measured using a Nanodrop 2000 spectrophotometer (Thermo Scientific, Wilmington, DE). Total RNA was reverse transcribed using the PrimeScriptRT Master Mix (TaKaRaBioTechnology), and PCR was performed using Premix Taq (TaKaRaTaq Version 2.0 plus dye, TaKaRaBioTechnology). The mRNA specific primers of CTR1, CTR2, ATP7A and ATP7B were purchased from Genscript Corp. (Nanjing, China). The PCR products were separated by electrophoresis in 2% agarose gels and were detected using the AlphaImager HP system (Alpha Innotech, San Leandro, CA, USA).

### Quantitative reverse transcription-PCR (qRT-PCR)

Total RNA was extracted by the RNAiso Plus (TaKaRaBio Technology, Dalian, China) following the manufacturer’s protocol. For mRNA quantative analysis, total RNA was reverse transcribed using the PrimeScriptTM RT MasterMix (TaKaRaBio Technology, Dalian, China), and qPCR was performed using SYBRPremix Ex Taq II (TaKaRaBio Technology, Dalian, China). The mRNA specific primers of CTR1, CTR2, ATP7A and ATP7B were purchased from Genscript Corp (Nanjing, China). All qPCR was performed with the Applied Biosystems 7300 Real Time PCR System (Applied Biosystems, Foster City, CA, USA) according to manufacturer’s instructions. Expression of mRNA was defined from the threshold cycle, and relative expression levels were calculated using the 2^-△△Ct^ method after normalization with reference to the expression of GAPDH. Primer sequences are listed below:

GAPDH; 5’- CAAGGTCATCCATGACAACTTTG-3’ (forward)

5’- GTCCACCACCCTGTTGCTGTAG -3’ (reverse)

CTR1: 5’-GGGGATGAGCTATATGGACTCC-3’ (forward) 5’-TCACCAAACCGGAAAACAGTAG-3’ (reverse);

CTR2; 5’-ATACAGCGGTGCTTCTGTTTG-3’ (forward) and 5’-GGTTGGCAGGTTCACCAGTA-3’ (reverse); 5’-TGACCCTAAACTACAGACTCCAA-3’ (forward) and 5’-CGCCGTAACAGTCAGAAACAA-3’ (reverse) for ATP7A; 5’-GCCAGCATTGCAGAAGGAAAG-3’ (forward) and 5’-TGATAAGTGATGACGGCCTCT-3’ (reverse) for ATP7B.

### Western blotting

After the indicated treatment, the cells were harvested and lysed by RIPA buffer (KeyGENBioTECH, Nanjing, China). The protein was extracted and its concentration was measured by BCA Protein Assay Kit (Beyotime, Shanghai, China). SDS-PAGE was used to separate 60 μg of protein. Then followed by transfer onto a PVDF membrane (Millipore Corporation, MA, USA). Membranes were blocked for 1 h at room temperature with 5% non-fat dry milk in Tris-Buffered-Saline with Tween (TBST), followed by incubation overnight at 4°C with specific antibodies. After being washed 3 times for 5 min with TBST, the membrane was then incubated with appropriate secondary antibodies for 1 h at room temperature. After extensive washing with TBST, proteins were visualized by the SuperSignal West Pico (Thermo Scientific, Wilmington, DE, USA). Antibodies used include: anti-CTR1 (1:1000, Santa Cruz Biotechnology, CA, USA), anti-β-actin (1:1000, BOSTER, Wuhan, China), HRP-Conjugated AffiniPure Goat Anti-Rabbit IgG (1:2000, ZSGB-BIO, Beijing, China), HRP-Conjugated AffiniPure Goat Anti-Mouse IgG (1:2000, ZSGB-BIO, Beijing, China).

### Ethics statement

This study was carried out strictly with the recommendations in the Guide for the Care and Use of Laboratory Animals of the National Institutes of Health. The protocol was approved by the Committee on the Ethics of Animal Experiments of Nanjing medical university (Permit Number: 2120474).

### Xenograft mouse model

Female BALB/c nude mice, 3–5 weeks of age, were purchased from Shanghai Animal Laboratory Center (Shanghai, China) and maintained in appropriate sterile filter-capped cages with lights turned on at 8:30 am in the Experimental Animal Center at Nanjing Medical University. The temperature and humidity was 22 ± 1°C and 55 ± 5%. We put wood shavings, bedding and a cardboard tube in all cages for environmental enrichment. All mice were observed every day to monitor abnormal behavior, such as inability to eat or drink, no response when stimulated, or unable to run away when touched. Exponentially growing OVCAR3 cells (5 × 10^6^) were injected subcutaneously into the dorsum of the mice. After tumor transplantation for 1 week, the body weight and the tumor size were recorded twice a week. The length and width of tumor were measured using a caliper, and the volumes were calculated by the following formula: volume (mm^3^) = length × width × width/2. At the 18th day after transplantation, 32 mice were randomized into 4 groups (6 mice of control group and 8 mice of the other group) and treated as follows: control (normal saline, 0.1ml/10g), EGCG (20 mg/kg), cDDP (5 mg/kg), EGCG (20 mg/kg) and cDDP (5 mg/kg). EGCG was administered twice a week and cDDP was given once a week by intraperitoneal injection. After treatment for 4 weeks, xenograft tumors and kidney tissue were isolated from mice. A portion of the tumors and kidney tissue were fixed in 4% paraformaldehyde for histological study, and the rest were prepared for further experiments. We tried our best to minimize suffering of the Female BALB/c nude mice. No mice were sacrificed before the end of the experiment and the mice were euthanized by cervical dislocation.

### Renal damage indicators

Renal damage was measured with blood urea nitrogen (BUN) and plasma creatinine. BUN was measured by diagnostic kits (Jiancheng Bioengineering Institute, Nanjing, China) according to the assay kit protocols. We used urease methods to measure the BUN and picric acid methods to test the serum creatintine.

### Statistical methods

All data were presented as the mean ± standard deviation (SD) of at least three independent experiments. Western blotting was quantified using Image J software. Statistical analysis was performed using the student’s *t* test and One-Way ANOVA. The data were considered statistically significant when the *P* value was less than 0.05. (*P<0.05, **P<0.01)

## Results

### EGCG enhances the sensitivity of the ovarian cancer cells to cDDP

**Fig 1:**
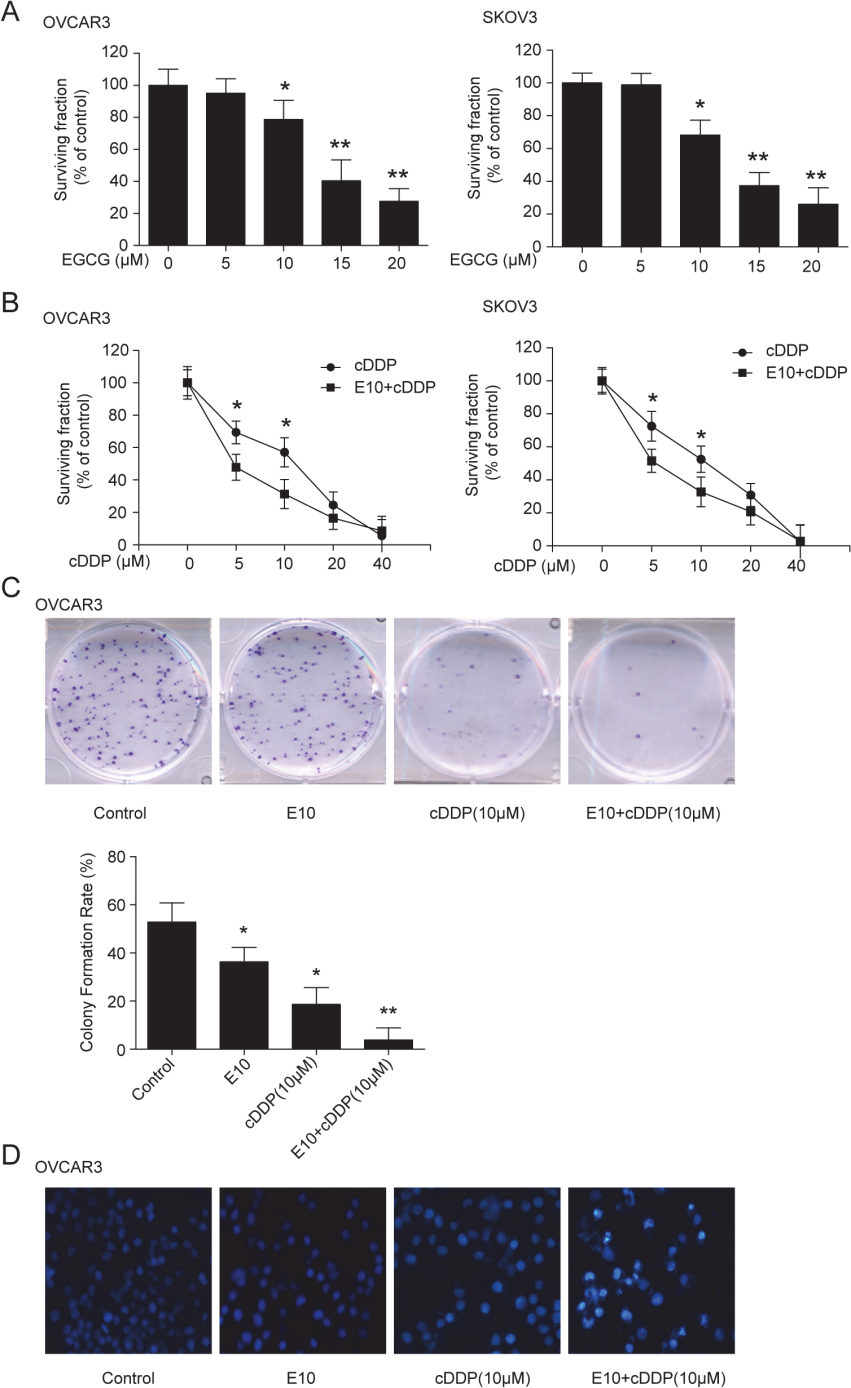
Effect of EGCG on the sensitivity of the ovarian cancer to cDDP. (A) Effect of EGCG on ovary cancer cells survival fraction. OVCAR3 and SKOV3 cells were treated with the indicated concentrations of EGCG for 24 h, then followed by MTT assay to detect cell the survival fraction. (B) Effect of the combination of EGCG and cDDP on cell survival fraction. The cells were treated with indicated concentration of cDDP alone, or in combination with the EGCG (10 μM, shown as E10), and followed by MTT assay. (C) EGCG in combination with cDDP repressed colony formation. OVCAR3 cells were treated with 10 μM of EGCG alone or in combination of 10 μM of cDDP for 48 h. When the colonies formed two weeks later, colony formation assay was carried out. (D) The combination of EGCG and cDDP on cells apoptosis, Hoechst 33258 staining was used to detect apoptosis caused by the indicated treatments. (*P<0.05, **P<0.01)

To determine the suitable concentration of EGCG on cells survival, ovary cancer OVCAR3 and SKOV3 cells were treated with indicated doses of EGCG. As Fig 1A showed, from 10 μM, EGCG caused significantly inhibition of the cells growth. Therefore, 10 μM EGCG was chosen for combination with cDDP. To determine the effect of EGCG on cDDP sensitivity, OVCAR3 and SKOV3 cells were treated with 10μM EGCG and various concentration of cDDP, either alone or in combination. As shown in Fig 1B, cDDP alone inhibited the growth of the ovary cancer cells in a dose-dependent manner, while the combination of EGCG and cDDP exhibited a greater inhibition. In OVCAR3 cells, the IC_50_ (mean ± SEM) was 9.49 ± 0.13 μM for cDDP alone and 3.48 ± 0.24 μMcDDP for the combination. The combination decreased IC_50_ for 2.7-fold in SKOV3 cells. The IC_50_ had a 1.6-fold decrease in the combination of EGCG and cDDP (6.14 ± 0.38 μM), compared with cDDP (9.65 ± 0.56 μM) only treatment.

To further confirm the effect of EGCG and cDDP on cell growth, a colony formation assay was conducted. EGCG or cDDP inhibited colony growth, while the combination of the two conferred a more pronounced repression (Fig 1C). The possible induction of apoptosis in OVCAR3 cells by EGCG and cDDP was investigated by Hoechst 33258 staining. The combination of the two agents showed significant apoptosis in OVCAR3 cells (Fig 1D).

### EGCG increases cDDP and DNA-Pt adducts accumulation in cells

**Fig 2:**
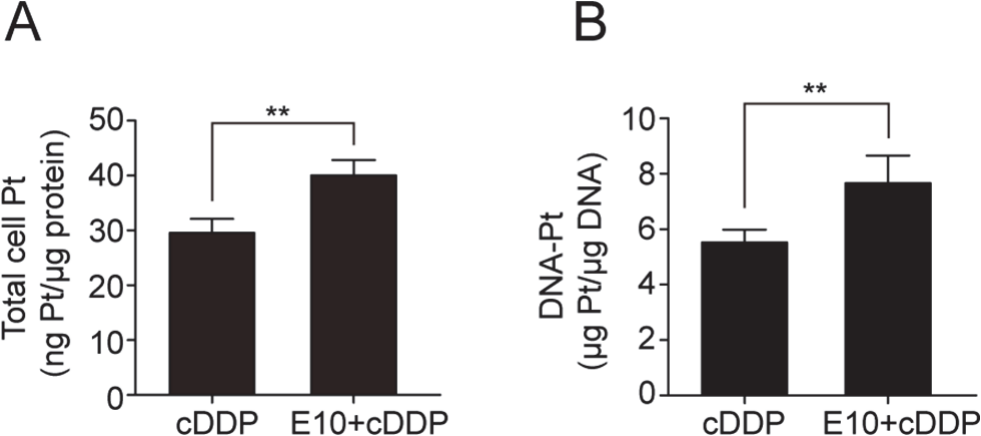
Effects of EGCG on Pt and DNA-Pt adducts accumulation in the cells. OVCAR3 cells were treated with/without 10μM EGCG and 30 μMcDDP for 4 h, and then followed by ICP-MC assay. (A) Whole-cell Pt accumulation. (B) DNA-Pt adducts accumulation. (**P<0.01)

To investigate whether changes in sensitivity to cDDP were resulted from changes in cellular cDDP and DNA-Pt accumulation, the whole-cell content of Pt was measured by ICP-MS. Fig 2A showed that the OVCAR3 cells exposed to 30 μM of cDDP for 4 h accumulated 29.57±2.52 ng of Pt per 1 μg protein; whereas the cells treated with combined EGCG and cDDP accumulated 40.04±2.78 ng of Pt per 1 μg protein, representing a 35% elevation as compared with the cDDP group (*P*<0.01).

The primary mechanism of action of Pt-containing drugs is thought to format DNA-Pt adducts and cause cytotoxicity. Thus, the DNA-Pt adduct concentration was measured in DNA extracted from cells with 30μMof cDDP or cDDP combined with 10 μM of EGCG. The combination of the two agents caused a 42% increase in the DNA-Pt accumulation (7.66±0.70 versus 5.41±0.46 μg Pt/μg DNA; *P*<0.01) (Fig 2B). Therefore, the observation of EGCG enhancing cDDP sensitivity might be partly explained by a proportional increase in the amount of the cDDP and DNA-Pt accumulation in the cells.

### EGCG promotes the expression of CTR1 but not the other copper transporters

**Fig 3:**
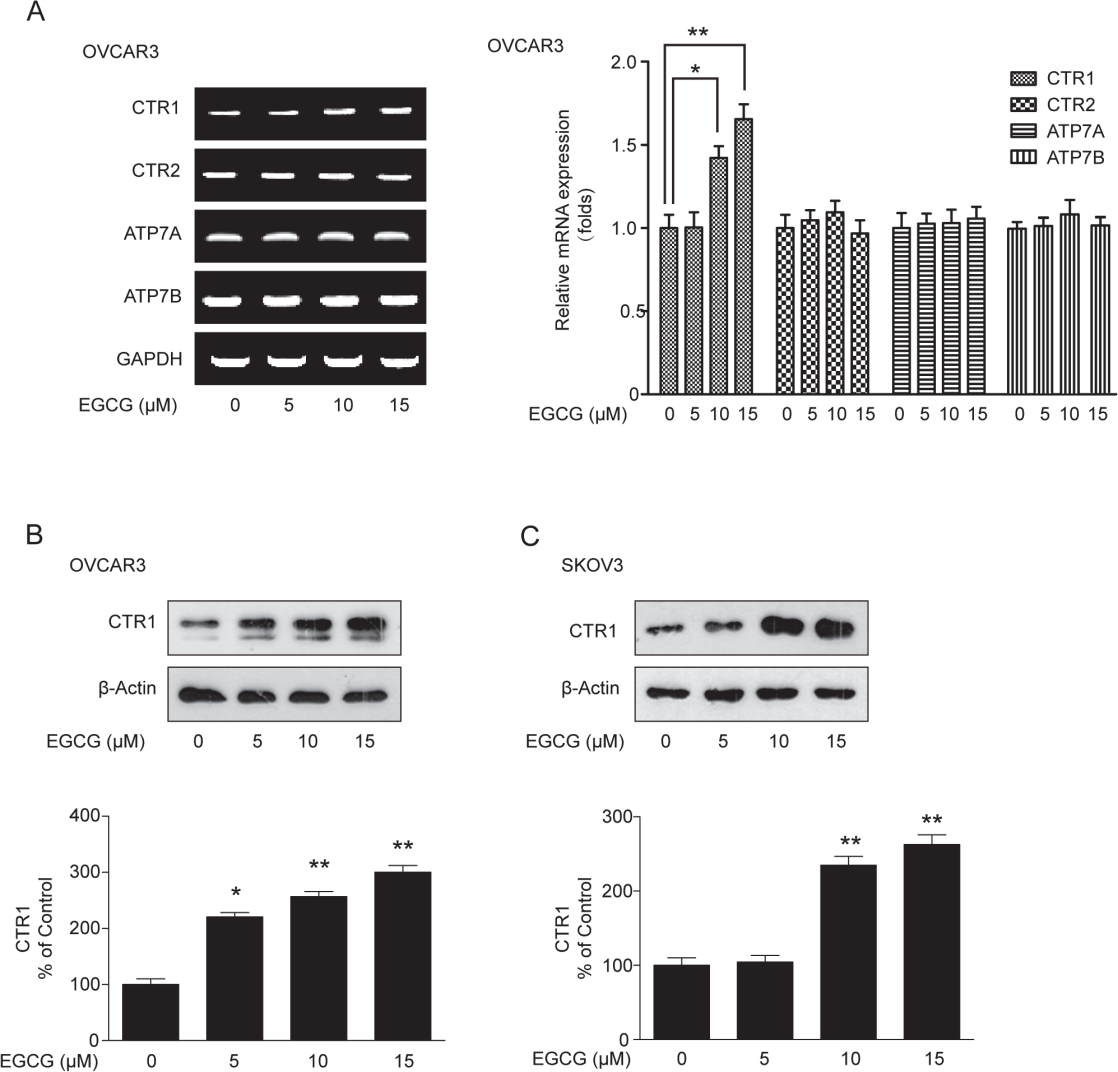
Effect of EGCG on the expression of copper transporters. (A) Effect of EGCG on mRNA expression of CTR1, CTR2, ATP7A and ATP7B. After OVCAR3 cells were treated with indicated concentrations of EGCG for 24h, RT-PCR and qPCR were carried out to measure the CTR1 mRNA expression. (B and C) Effect of EGCG on CTR1 protein expression in OVCAR3 and SKOV3 cells. The cells were treated with 10 μM EGCG for 24 h, then followed by western blot analysis. The bands were quantified with Image J software. (*P<0.05, **P<0.01)

Given the fact that the copper transporters can partly influence the sensitivity of ovarian cancer cells to cDDP, the relative expression of copper transporters was quantified by RT-PCR and qPCR. As shown in Fig 3A, CTR1 mRNA was elevated after EGCG treatment in OVCAR3 cells. However, there were no significant changes in mRNA expression of CTR2, ATP7A and ATP7B upon EGCG treatment. Next, we focused on whether EGCG could affect CTR1 protein expression. As shown in Fig 3B, EGCG was able to induce CTR1 protein expression in a dose-dependent manner either in OVCAR3 and SKOV3 cells (Fig 3C). Taken together, EGCG can induce CTR1 expression in ovary cancer cells.

### Knock-down of CTR1 changes the sensitivity of cells to cDDP

**Fig 4:**
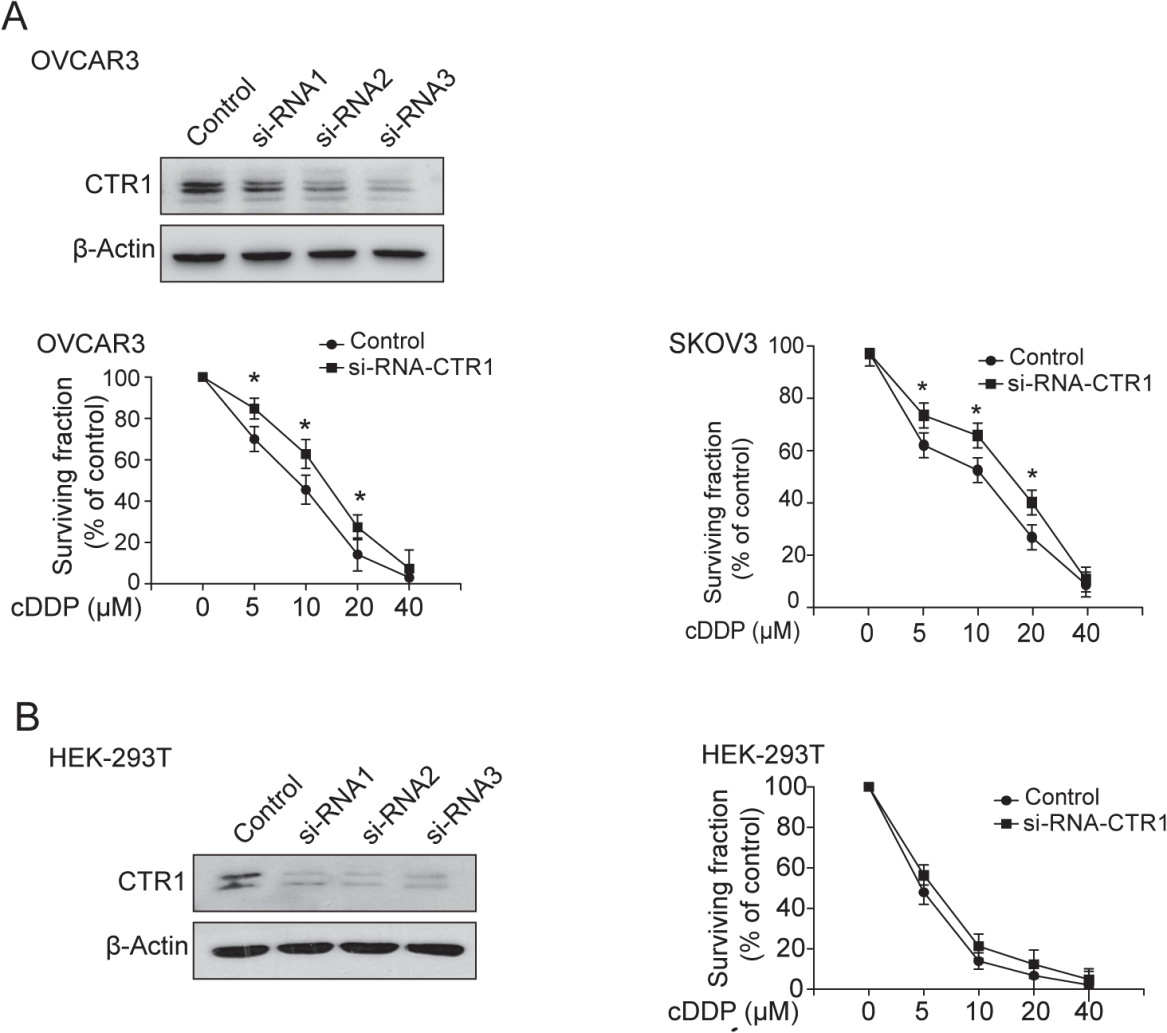
Effect of knock-down of CTR1 on the sensitivity of cells to cDDP. (A) Effect of knock-down of CTR1 on the sensitivity of ovary cancer cells to cDDP. OVCAR3 cells were transfected with three company sythetic si-RNAs or siRNA control and the western blost analysis showed si-RNA 3 exhibiting the best effect. After transfected with si-RNA3 or siRNA control, OVCAR3 and SKOV3 cells were treated with cDDP at various doses for 48 h and the cell survival fraction was detected by MTT assay. (B) Embryonic kidney HEK-293 cells were tranfected with human CTR1 si-RNA3 or siRNA control. Then the cells were exposed by indicated doses of cDDP for 48 h and the cell survival fraction was detected by MTT assay. (*P<0.05).

To further confirm the relationship between CTR1 and cDDP sensitivity, OVCAR3 cells were transfected with CTR1 siRNAs. The si-RNA3 shows the best knockdown effect. The expression of CTR1 (Fig 4A and 4B) and the survival fraction of cells responding to cDDP were measured. As shown in Fig 4A and 4B, knockdown of CTR1 decreased the expression of CTR1 and lowered the sensitivity of ovarian cancer OVCAR3 and SKOV3 cells to cDDP. Interestingly, knockdown of CTR1 had no statistically significant effect in human embryonic kidney HEK-293T cells, though the expression of CTR1 was almost completely inhibited. These results indicated that the influence of CTR1 to cDDP might be different between cell types.

### EGCG inhibits the degradation of CTR1 induced by cDDP

**Fig 5:**
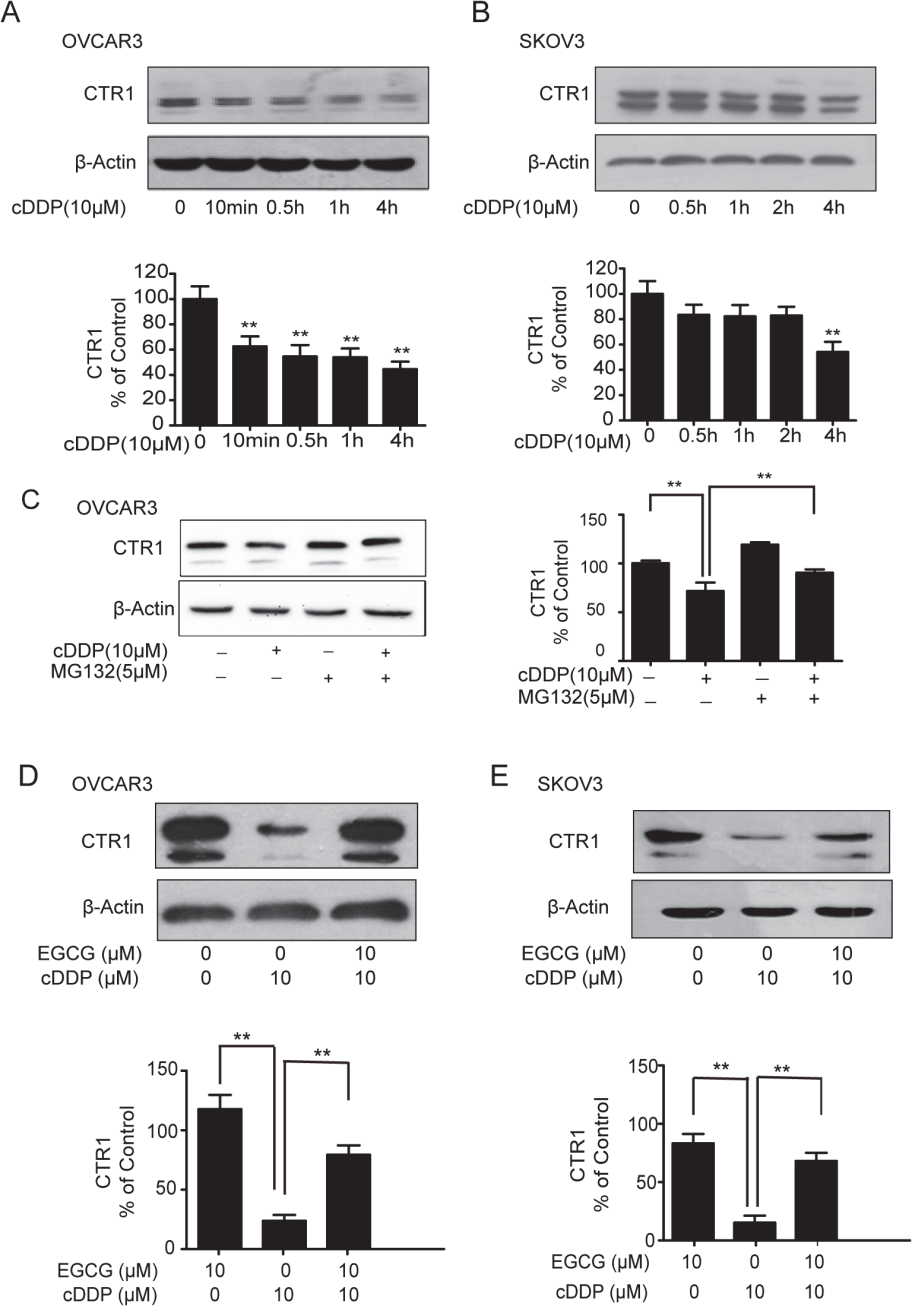
EGCG inhibits the degredation of CTR1 induced by cDDP. (A, B) The effect of cDDP on the expression of CTR1 in OVCAR3 and SKOV3 cells. The cells were treated with 10 μM cDDP for the indicated time and CTR1 protein expression were detected by western blot analysis. (C)The effect of MG132 on the degradation of CTR1. After OVCAR3 cells were pretreated with 5 μM MG132 for 10h, the cells were incubation with 10 μM cDDP for 14 h. Then followed by western blot analysis. (D, E) The effect of EGCG on cDDP-trigged decrease of CTR1. The OVCAR3 and SKOV3 cells were treated with/without 10μM EGCG in the presence/absence of 10μMcDDP for 24 h. CTR1 protein expression was detected. The bands were quantified with Image J software. (*P<0.05, **P<0.01)

Previous studies have shown that, even at lower concentrations, cDDP can induce rapid degradation of CTR1 [15]. As shown in Fig 5A and 5B, 10 μM of cDDP decreased the expression of CTR1 in a time-dependent manner in OVCAR3 and SKOV3 cells. To determine the contribution of proteasomal degradation to the down-regulation of CTR1 induced by cDDP, OVCAR3 cells were incubated with cDDP alone or in combination with MG132 (a proteasome inhibitor), western blot analysis indicated that MG132 blocked the degradation of CTR1 (Fig 5C). Since EGCG could induce the expression of CTR1, we next determined whether EGCG could prevent the degradation of CTR1 induced by cDDP. Fig 5D and 5E showed that EGCG inhibited the rapid degradation of CTR1 caused by cDDP treatment in ovarian cancer OVCAR3 and SKOV3 cells.

### EGCG enhances the efficacy of cDDP in inhibiting xenograft tumor growth and attenuates the nephrotoxicity of cDDP in vivo

**Fig 6:**
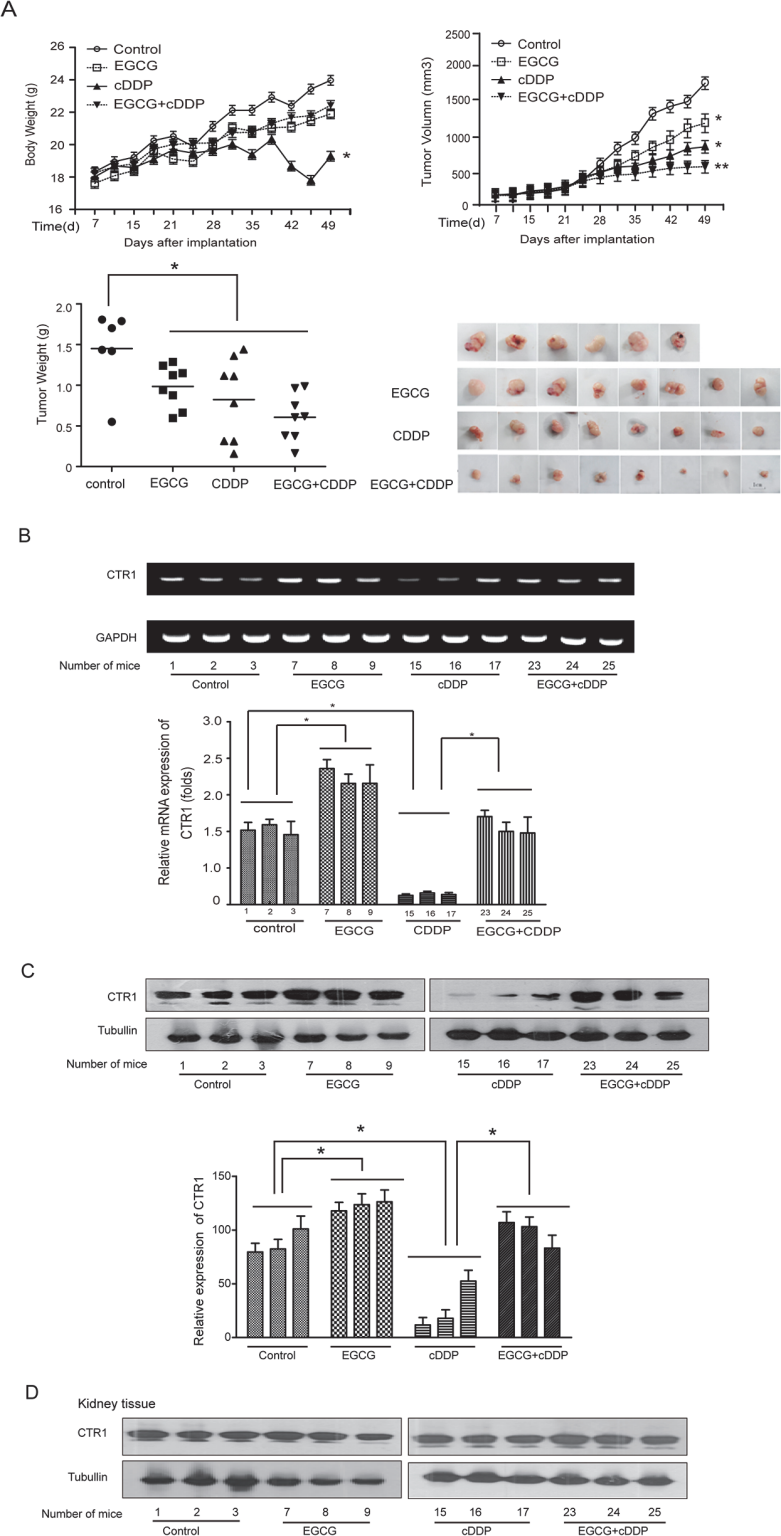
EGCG enhances the efficacy of cDDP on tumor responsiveness and attenuates the nephrotoxicity induced by cDDP in vivo. Four groups (control, EGCG, cDDP and EGCG+cDDP) were set up. Except there were 6 mice in control group, there were 8 mice for each of the other groups. The body weight (A) and the tumor size (A) were measured twice a week. (B) The mRNA expression of the CTR1 in tumor tissues was measured by RT-PCR and real qPCR. (C) The expression of CTR1 in tumor tissue was assessed by western blotting. (D) The expression of CTR1 in kidney tissue was measured by western blotting. The bands were quantified by Image J software. (*P<0.05, **P<0.01)

To further investigate whether the enhanced efficacy of cDDP by EGCG could occur *in vivo*, we established an OVCAR3 ovarian xenograft model in nude mice as described in the Materials and Methods section. The body weight and tumor size were measured twice weekly. The body weights of the cDDP-treated groups were significantly lower compared with the other groups (Fig 6A). The administration of EGCG exhibited a protective effect against the cDDP-induced weight loss. As shown in Fig 6A, EGCG or cDDP alone suppressed the growth of tumor, while EGCG combined with cDDP had a stronger inhibitory effect than other treatments, which was consistent with the results *in vitro* experiments.

The mRNA and protein expression of CTR1 were measured in tumor tissues (Fig 6B). As it was shown, EGCG increased the mRNA expression of CTR1 significantly. It also appeared that the protein expression of the CTR1 was significantly decreased by cDDP, but enhanced by EGCG and returned to a normal level by EGCG combined with cDDP (Fig 6C).

The renal damage indicators such as kidney weight, blood urea nitrogen (BUN) and plasma creatinine were used to evaluate possible renal damage. The mice treated with cDDP, their kidney weights, BUN and creatinine were significantly higher than those of other groups. Administration of EGCG reduced the levels of these cDDP-induced kidney toxicity indicators. Treatment with EGCG alone did not affect these indicators (Table 1). All these results demonstrated that EGCG could partly protect against nephrotoxicity induced by cDDP *in vivo*. To investigate whether the protective effect of EGCG on cDDP-induced nephrotoxicity was associated with the uptake of cDDP, we measured the relative expression of CTR1 in kidney tissues. The expression of CTR1 had no significant changes in kidney tissues after the indicated treatments (Fig 6D).

**Table 1:** Changes of kidney weight and kidney injury indicators in various groups.

Experimental group	Conrtol (n = 6)	EGCG (n = 8)	cDDP (n = 8)	EGCG+cDDP (n = 8)
Kidney weight/body weight(%)	1.32±0.12	1.49±0.09	1.69±0.25*	1.46±0.23
BUN(mmol/l)	15.26±1.96	16.04±1.28	19.46±2.77*	16.5±1.76
Creatinine(μmol/l)	2.02±0.97	2.69±0.94	3.79±1.57*	2.38±0.88

Values are presented as mean ± SD. The “n” means mice number.

* values differ significantly from control (*p<0*.*05*)

## Discussion

In the current study, we systematically investigated the effects of EGCG on copper transporters and the uptake and efflux of cDDP in ovary cancer cells and xenograft mice. To our knowledge, this is the first report demonstrating that EGCG increased CTR1 expression *in vitro* and *in vivo*. Also, EGCG prevented the degradation of CTR1 induced by cDDP. EGCG treatment in ovarian cancer resulted in a significant increase in sensitivity to cDDP, which might be due to the up-regulation of CTR1, increased accumulation of cDDP and DNA-Pt adducts. Our findings provided a mechanism for the strategy of using EGCG as an adjuvant for ovarian cancer therapy.

Previous studies showed that the expression of CTR1 could be regulated under different copper stress at the transcriptional and post-translational levels. At the transcriptional level, the mRNA of CTR1 was regulated by the transcription factor Sp1 [33]. The zinc finger (ZF) domain of Sp1 was found to function as sensors of copper stress [33], and there existed a Copper-hCtr1-Sp1 Inter-Regulatory Loop in copper homeostasis [34]. Post-translational mechanisms have also been related to copper stress. Copper chelator or copper-lowering agents were shown to induce CTR1 expression and enhance the uptake of cDDP by cells and their sensitivity to cDDP [10, 35, 36]. High concentrations of copper and cDDP induced endocytosis or degradation of CTR1 [15,37,38]. The mechanism through which EGCG mediates CTR1 expression is still not understood. More studies on this topic are needed.

In the present study, cDDP(in 10 μM) caused rapid degradation of CTR1 (Fig 5A and 5B). Multiple lines of evidence indicated that cDDP-induced degradation of CTR1 involves ubiquitination and proteosomal degradation. A proteasome inhibitor (bortezomib, actacystin or MG132) given together with cDDP blocked cDDP-induced loss of CTR1 and enhanced delivery of cDDP [39–41]. Combination of cDDP with EGCG or MG132 both inhibited the degradation of CTR1 induced by cDDP (Fig 5C, 5D and 5E). It was reported that EGCG could inhibit the proteasome activity [22, 42]. The inhibition of ubiquitin-proteasome by EGCG contributed to overcome resistance to various chemotherapeutic drugs and enhance the effect of chemotherapy [43]. Thus, the inhibition of cDDP-induced degradation of CTR1 by EGCG might be due to its action on ubiquitin-proteasome.

Nephrotoxicity is the major complication of the clinical application of cDDP and limits its long-term usage. Previous studies have reported that EGCG could prevent the deleterious effects of cDDP [44–46]. Our study also indicated that EGCG had a protective effect against cDDP-induced nephrotoxicity (Table 1). One of the reasons could be that EGCG didn’t change CTR1 expression in kidney tissues (Fig 6), while EGCG induced CTR1 expression in ovarian cancer cells and tumor tissues (Figs 3 and 6). Knockdown of CTR1 in human embryonic kidney HEK-293T cells had on significant changes to cDDP sensitivity (Fig 4B). Why EGCG did not change CTR1 expression in kidney cells and tissues is interesting and needs further investigation.

The protective effect of EGCG against nephrotoxicity may be also due to its antioxidant and anti-inflammation activities as suggested previously [44–46]. Recent studies suggested that organic cation transporter 2 (OCT2) was a target for protective interventions in cDDP-induced nephrotoxicity [47–49]. Our preliminary data showed that EGCG could regulate OCT2 as well, and this is under further investigation.

In summary, this is the first report that EGCG could induce the expression of CTR1, leading to the accumulation of cellular cDDP and cDDP-DNA adducts and enhanced sensitivity of ovarian cancer cells to cDDP. The findings provided a novel mechanism for exploring new strategies for green tea polyphenol EGCG as an adjuvant for the treatment of ovarian cancer.

## References

[1] Garces AH, Dias MS, Paulino E, Ferreira CG, de Melo AC. Treatment of ovarian cancer beyond chemotherapy: Are we hitting the target? Cancer Chemother Pharmacol 2015; 75:221–34. 10.1007/s00280-014-2581-y 25212538

[2] Mei L, Chen H, Wei DM, Fang F, Liu GJ, Xie HY, et al Maintenance chemotherapy for ovarian cancer. Cochrane Database Syst Rev 2013; 6:D7414. 10.1002/14651858.CD007414.pub3 PMC6457821 23813336

[3] Jamieson ER, Lippard SJ. Structure, Recognition, and Processing of Cisplatin-DNA Adducts. Chem Rev 1999; 99:2467–98. 11749487 10.1021/cr980421n

[4] Galluzzi L, Senovilla L, Vitale I, Michels J, Martins I, Kepp O, et al Molecular mechanisms of cisplatin resistance. Oncogene 2012; 31:1869–83. 10.1038/onc.2011.384 21892204

[5] Safaei R. Role of copper transporters in the uptake and efflux of platinum containing drugs. Cancer Lett 2006; 234:34–39. 16297532 10.1016/j.canlet.2005.07.046

[6] Hall MD, Okabe M, Shen DW, Liang XJ, Gottesman MM. The role of cellular accumulation in determining sensitivity to platinum-based chemotherapy. Annu Rev Pharmacol Toxicol 2008; 48:495–535. 17937596 10.1146/annurev.pharmtox.48.080907.180426

[7] Tsigelny IF, Sharikov Y, Greenberg JP, Miller MA, Kouznetsova VL, Larson CA, et al An all-atom model of the structure of human copper transporter 1. Cell Biochem Biophys 2012; 63:223–34. 10.1007/s12013-012-9358-x 22569840 PMC3590913

[8] Lee J, Petris MJ, Thiele DJ. Characterization of mouse embryonic cells deficient in the ctr1 high affinity copper transporter. Identification of a Ctr1-independent copper transport system. J Biol Chem 2002; 277:40253–9. 12177073 10.1074/jbc.M208002200

[9] Ishida S, Lee J, Thiele DJ, Herskowitz I. Uptake of the anticancer drug cisplatin mediated by the copper transporter Ctr1 in yeast and mammals. Proc Natl Acad Sci U S A 2002; 99:14298–302. 12370430 10.1073/pnas.162491399 PMC137878

[10] Ishida S, McCormick F, Smith-McCune K, Hanahan D. Enhancing tumor-specific uptake of the anticancer drug cisplatin with a copper chelator. Cancer Cell 2010; 17(6):574–83. 10.1016/j.ccr.2010.04.011 20541702 PMC2902369

[11] Noordhuis P, Laan AC, van de Born K, Losekoot N, Kathmann I, Peters GJ. Oxaliplatin activity in selected and unselected human ovarian and colorectal cancer cell lines. Biochem Pharmacol 2008; 76(1):53–61. 10.1016/j.bcp.2008.04.007 18508032

[12] Holzer AK, Samimi G, Katano K, Naerdemann W, Lin X, Safaei R, et al The copper influx transporter human copper transport protein 1 regulates the uptake of cisplatin in human ovarian carcinoma cells. Mol Pharmacol 2004; 66: 817–23. 15229296 10.1124/mol.104.001198

[13] Lin X, Okuda T, Holzer A, Howell SB. The copper transporter CTR1 regulates cisplatin uptake in Saccharomyces cerevisiae. Mol Pharmacol 2002; 62:1154–9. 12391279 10.1124/mol.62.5.1154

[14] Larson CA, Blair BG, Safaei R, Howell SB. The role of the mammalian copper transporter 1 in the cellular accumulation of platinum-based drugs. Mol Pharmacol 2009; 75:324–30. 10.1124/mol.108.052381 18996970 PMC2684896

[15] Holzer AK, Katano K, Klomp LW, Howell SB. Cisplatin rapidly down-regulates its own influx transporter hCTR1 in cultured human ovarian carcinoma cells. Clin Cancer Res 2004; 10:6744–9. 15475465 10.1158/1078-0432.CCR-04-0748

[16] Safaei R, Maktabi MH, Blair BG, Larson CA, Howell SB. Effects of the loss of Atox1 on the cellular pharmacology of cisplatin. J Inorg Biochem 2009; 103:333–41. 10.1016/j.jinorgbio.2008.11.012 19124158 PMC2919289

[17] Blair BG, Larson CA, Adams PL. Copper transporter 2 regulates endocytosis and controls tumor growth and sensitivity to cisplatin in vivo. Mol Pharmacol 2011; 79:157–66. 10.1124/mol.110.068411 20930109 PMC3014285

[18] Blair BG, Larson CA, Safaei R, Howell SB. Copper transporter 2 regulates the cellular accumulation and cytotoxicity of Cisplatin and Carboplatin. Clin Cancer Res 2009; 15:4312–21. 10.1158/1078-0432.CCR-09-0311 19509135 PMC2862640

[19] Blair BG, Larson CA, Adams PL, Abada PB, Safaei R, Howell SB. Regulation of copper transporter 2 expression by copper and cisplatin in human ovarian carcinoma cells. Mol Pharmacol 2010; 77:912–21. 10.1124/mol.109.062836 20194531 PMC2879916

[20] Samimi G, Varki NM, Wilczynski S, Safaei R, Alberts DS, Howell SB. Increase in expression of the copper transporter ATP7A during platinum drug-based treatment is associated with poor survival in ovarian cancer patients. Clin Cancer Res 2003; 9:5853–9. 14676106

[21] Nakayama K, Kanzaki A, Ogawa K, Miyazaki K, Neamati N, Takebayashi Y. Copper-transporting P-type adenosine triphosphatase (ATP7B) as a cisplatin based chemoresistance marker in ovarian carcinoma: comparative analysis with expression of MDR1, MRP1, MRP2, LRP and BCRP. Int J Cancer 2002; 101(5):488–95. 12216079 10.1002/ijc.10608

[22] Yang CS, Wang X, Lu G, Picinich SC. Cancer prevention by tea: animal studies, molecular mechanisms and human relevance. Nat Rev Cancer 2009; 9: 429–39. 10.1038/nrc2641 19472429 PMC2829848

[23] Yang CS, Feng Q. Chemo/Dietary prevention of cancer: perspectives in China. J Biomed Res2014; 28:447–55 10.7555/JBR.28.20140079 25469113 PMC4250523

[24] Yang H, Landis-Piwowar K, Chan TH, Dou QP. Green tea polyphenols as proteasome inhibitors: implication in chemoprevention. Curr Cancer Drug Targets 2011; 11:296–306. 21247384 10.2174/156800911794519743 PMC3304300

[25] Lecumberri E, Dupertuis YM, Miralbell R, Pichard C. Green tea polyphenol epigallocatechin-3-gallate (EGCG) as adjuvant in cancer therapy. Clin Nutr 2013; 32:894–903. 10.1016/j.clnu.2013.03.008 23582951

[26] Hagen RM, Chedea VS, Mintoff CP, Bowler E, Morse HR, Ladomery MR. Epigallocatechin-3-gallate promotes apoptosis and expression of the caspase 9a splice variant in PC3 prostate cancer cells. Int J Oncol 2013; 43:194–200. 10.3892/ijo.2013.1920 23615977

[27] Chen H, Landen CN, Li Y, Alvarez RD, Tollefsbol TO. Enhancement of Cisplatin-Mediated Apoptosis in Ovarian Cancer Cells through Potentiating G2/M Arrest and p21 Upregulation by Combinatorial EpigallocatechinGallate and Sulforaphane. J Oncol 2013; 2013:872957 10.1155/2013/872957 23476648 PMC3588178

[28] Chan MM, Soprano KJ, Weinstein K, Fong D. Epigallocatechin-3-gallate delivers hydrogen peroxide to induce death of ovarian cancer cells and enhances their cisplatin susceptibility. J Cell Physiol 2006; 207:389–96. 16402374 10.1002/jcp.20569

[29] Shervington A, Pawar V, Menon S, Thakkar D, Patel R. The sensitization of glioma cells to cisplatin and tamoxifen by the use of catechin. Mol Biol Rep 2009; 36:1181–6. 10.1007/s11033-008-9295-3 18581255

[30] Lee SH, Nam HJ, Kang HJ, Kwon HW, Lim YC. Epigallocatechin-3-gallate attenuates head and neck cancer stem cell traits through suppression of Notch pathway. Eur J Cancer 2013; 49:3210–8. 10.1016/j.ejca.2013.06.025 23876835

[31] Deng PB, Hu CP, Xiong Z, Yang HP, Li YY. Treatment with EGCG in NSCLC leads to decreasing interstitial fluid pressure and hypoxia to improve chemotherapy efficacy through rebalance of Ang-1 and Ang-2. Chin J Nat Med 2013; 11(3):245–53. 10.1016/S1875-5364(13)60023-0 23725836

[32] Zhou DH, Wang X, Feng Q. EGCG enhances the efficacy of cisplatin by downregulating hsa-miR-98-5p in NSCLC A549 cells. Nutr Cancer 2014; 66:636–44. 10.1080/01635581.2014.894101 24712372

[33] Song IS, Chen HH, AibaI, Hossain A, Liang ZD, Klomp LW, Kuo MT. Transcription factor Sp1 plays an important role in the regulation of copper homeostasis in mammalian cells. Mol Pharmacol 2008; 74:705–13. 10.1124/mol.108.046771 18483225 PMC2574735

[34] Liang ZD, Tsai WB, Lee MY, Savaraj N, Kuo MT. Specificity protein 1 (sp1) oscillation is involved in copper homeostasis maintenance by regulating human high-affinity copper transporter 1 expression. Mol Pharmacol 2012; 81:455–64. 10.1124/mol.111.076422 22172574 PMC3286298

[35] Chen HH, Kuo MT. Overcoming platinum drug resistance with copper-lowering agents. Anticancer Res 2013; 33:4157–61. 24122978 PMC4001922

[36] Liang ZD, Long Y, Tsai WB, Fu S, Kurzrock R, Gagea-Iurascu M, et al Mechanistic basis for overcoming platinum resistance using copper chelating agents. Mol Cancer Ther 2012; 11:2483–94. 10.1158/1535-7163.MCT-12-0580 22914438 PMC3496003

[37] Guo Y, Smith K, Lee J, Thiele DJ, Petris MJ. Identification of methionine-rich clusters that regulate copper-stimulated endocytosis of the human Ctr1 copper transporter. J Biol Chem 2004; 279(17):17428–33. 14976198 10.1074/jbc.M401493200

[38] Molloy SA, Kaplan JH. Copper-dependent recycling of hCTR1, the human high affinity copper transporter. J Biol Chem 2009; 284:29704–13. 10.1074/jbc.M109.000166 19740744 PMC2785602

[39] Holzer AK, Howell SB. The internalization and degradation of human copper transporter 1 following cisplatin exposure. Cancer Res 2006; 66:10944–52. 17108132 10.1158/0008-5472.CAN-06-1710

[40] Jandial DD, Farshchi-Heydari S, Larson CA, Elliott GI, Wrasidlo WJ, Howell SB. Enhanced delivery of cisplatin to intraperitoneal ovarian carcinomas mediated by the effects of bortezomib on the human copper transporter 1. Clin Cancer Res 2009; 15:553–60. 10.1158/1078-0432.CCR-08-2081 19147760 PMC2707998

[41] Mortenson MM, Schlieman MG, Virudachalam S, Bold RJ. Effects of the proteasome inhibitor bortezomib alone and in combination with chemotherapy in the A549 non-small-cell lung cancer cell line. Cancer Chemother Pharmacol 2004; 54:343–53. 15197486 10.1007/s00280-004-0811-4

[42] Nam S, Smith DM, Dou QP. Ester bond-containing tea polyphenols potently inhibit proteasome activity in vitro and in vivo. J Biol Chem 2001; 276:13322–30. 11278274 10.1074/jbc.M004209200

[43] Shen M, Chan TH, Dou QP. Targeting tumor ubiquitin-proteasome pathway with polyphenols for chemosensitization. Anticancer Agents Med Chem 2012; 12(8):891–901. 22292765 10.2174/187152012802649978 PMC3376667

[44] El-Mowafy AM, Salem HA, Al-Gayyar MM, El-Mesery ME, El-Azab MF. Evaluation of renal protective effects of the green-tea (EGCG) and red grape resveratrol: role of oxidative stress and inflammatory cytokines. Nat Prod Res 2011; 25:850–6. 10.1080/14786419.2010.533669 21462079

[45] El-Mowafy AM, Al-Gayyar MM, Salem HA, El-Mesery ME, Darweish MM. Novel chemotherapeutic and renal protective effects for the green tea (EGCG): role of oxidative stress and inflammatory-cytokine signaling. Phytomedicine 2010; 17:1067–75. 10.1016/j.phymed.2010.08.004 20851589

[46] Sahin K, Tuzcu M, Gencoglu H, Dogukan A, Timurkan M, Sahin N, et al Epigallocatechin-3-gallate activates Nrf2/HO-1 signaling pathway in cisplatin-induced nephrotoxicity in rats. Life Sci 2010; 87:240–5. 10.1016/j.lfs.2010.06.014 20619277

[47] Ciarimboli G, Deuster D, Knief A, Sperling M, Holtkamp M, Edemir B, Pavenstädt H, et al Organic cation transporter 2 mediates cisplatin-induced oto- and nephrotoxicity and is a target for protective interventions. Am J Pathol 2010; 176:1169–80. 10.2353/ajpath.2010.090610 20110413 PMC2832140

[48] Ciarimboli G, Ludwig T, Lang D, Pavenstädt H, Koepsell H, Piechota HJ, et al Cisplatin nephrotoxicity is critically mediated via the human organic cation transporter 2. Am J Pathol 2005; 167:1477–84. 16314463 10.1016/S0002-9440(10)61234-5 PMC1613191

[49] Yonezawa A, Masuda S, Nishihara K, Yano I, Katsura T, Inui K. Association between tubular toxicity of cisplatin and expression of organic cation transporter rOCT2 (Slc22a2) in the rat. Biochem Pharmacol 2005; 70:1823–31. 16242669 10.1016/j.bcp.2005.09.020

