# Correction: EGCG Enhances Cisplatin Sensitivity by Regulating Expression of the Copper and Cisplatin Influx Transporter CTR1 in Ovary Cancer

**DOI:** 10.1371/journal.pone.0132086

**Published:** 2015-06-29

**Authors:** Xuemin Wang, Pan Jiang, Pengqi Wang, Chung S. Yang, Xuerong Wang, Qing Feng

There are errors in Fig 6. The “Control” label in Fig 6A is missing and the tubulin bands in Fig 6D are mistakenly incorporated. Please view Fig 6 here.

**Fig 6:**
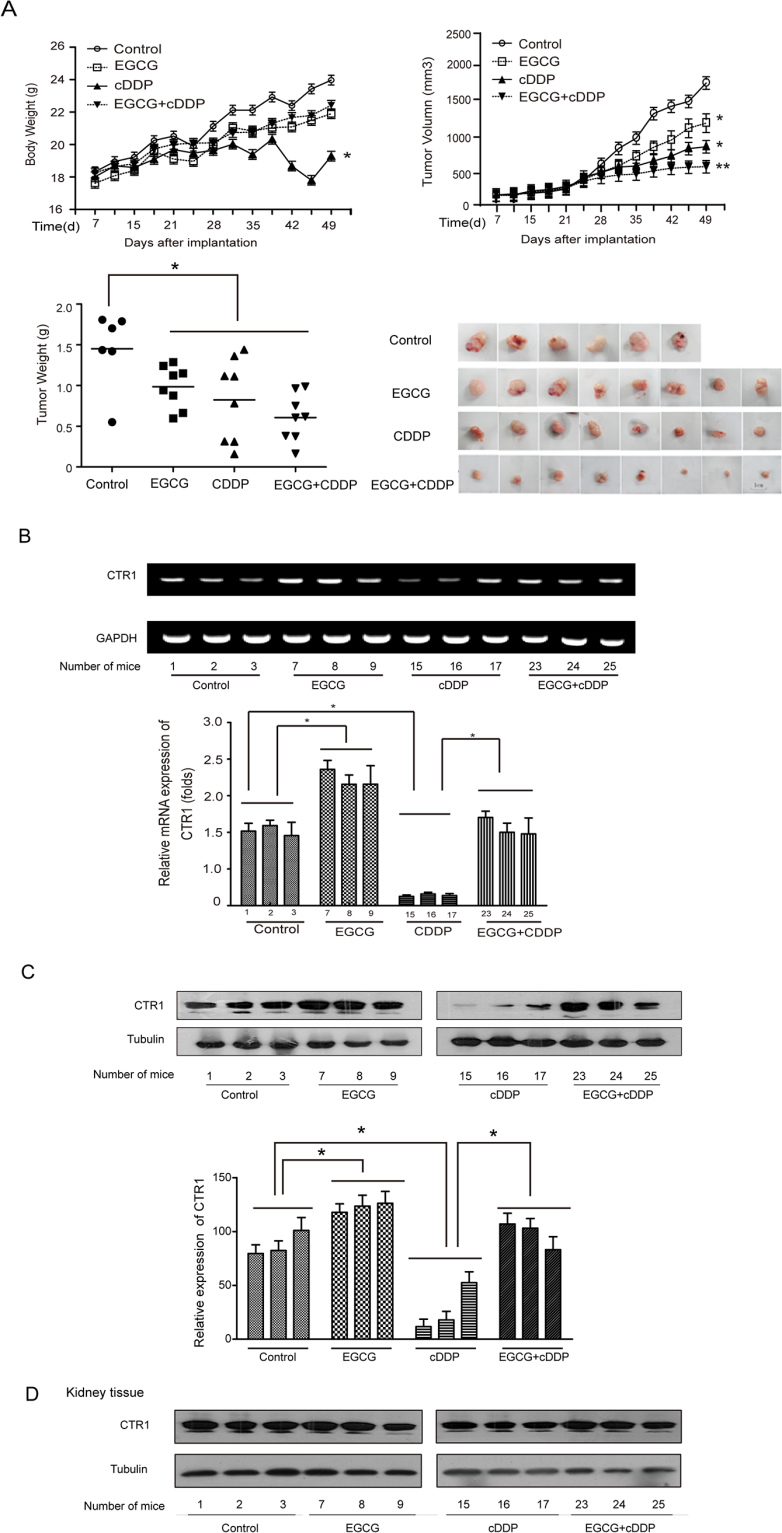
EGCG enhances the efficacy of cDDP on tumor responsiveness and attenuates the nephrotoxicity induced by cDDP in vivo. Four groups (control, EGCG, cDDP and EGCG+cDDP) were set up. Except there were 6 mice in control group, there were 8 mice for each of the other groups. The body weight (A) and the tumor size (A) were measured twice a week. (B) The mRNA expression of the CTR1 in tumor tissues was measured by RT-PCR and real qPCR. (C) The expression of CTR1 in tumor tissue was assessed by western blotting. (D) The expression of CTR1 in kidney tissue was measured by western blotting. The bands were quantified by Image J software. (*P<0.05, **P<0.01)
